# A randomized, double-blind, placebo-controlled study to assess efficacy of mirtazapine for the treatment of diarrhea predominant irritable bowel syndrome

**DOI:** 10.1186/s13030-021-00205-2

**Published:** 2021-02-03

**Authors:** Alireza Khalilian, Davoud Ahmadimoghaddam, Shiva Saki, Younes Mohammadi, Maryam Mehrpooya

**Affiliations:** 1 Department of Internal Medicine, School of Medicine, Hamadan University of Medical Sciences, Hamadan, Iran; 2 Department of Pharmacology & Toxicology, School of Pharmacy, Hamadan University of Medical Sciences, Hamadan, Iran; 3 Department of Clinical Pharmacy, School of Pharmacy, Hamadan University of Medical Sciences, Shahid Fahmideh Ave, Hamadan, 6517838678 Iran; 4 Modeling of Noncommunicable Diseases Research Center, School of Public Health, Hamadan University of Medical Sciences, Hamadan, Iran

**Keywords:** Irritable bowel syndrome, Diarrhea, 5-hydroxytryptamine, Mirtazapine

## Abstract

**Background:**

Ample evidence indicates the efficacy of serotonin type 3 (5-HT_3_) receptor antagonists in the treatment of patients with diarrhea-predominant irritable bowel syndrome (IBS-D). Mirtazapine is an atypical antidepressant with a well-known 5-HT_3_ receptor antagonist property. This study, therefore, was undertaken to investigate whether compared to placebo, mirtazapine would be efficacious and safe in the treatment of patients with IBS-D.

**Methods:**

From November 2019 until July 2020, 67 patients meeting Rome IV criteria for IBS-D were randomized in a double-blind fashion into either the mirtazapine treatment group (*n* = 34) or the placebo treatment group (*n* = 33). Patients started with mirtazapine 15 mg/day at bedtime for one-week; after which the dose was increased to 30 mg/day for an additional 7-week. Outcomes included changes in the total IBS symptom severity score (IBS-SSS), Hospital anxiety and depression scale score (HADS), and IBS Quality of Life. Additionally, changes in the diary-based symptoms scores including pain, urgency of defecation, bloating, stool frequency, and stool consistency based on the 7-point Bristol Stool Form Scale (BSFS), and a number of days per week with pain, urgency, diarrhea, or bloating, once during the 1-week run-in period, and once during the last week of treatment were recorded.

**Results:**

All analyses were performed on an Intention-to-Treat (ITT) analysis data set. The results showed compared to placebo, mirtazapine is more efficacious in decreasing the severity of IBS symptoms (*P*-value = 0.002). Further, at the end of the treatment period, all diary-derived symptoms except bloating showed significantly more improvement in the mirtazapine-treated subjects compared to the placebo-treated subjects. While was well-tolerated, mirtazapine also significantly improved the patients’ quality of life (*P*-value = 0.04) and anxiety symptoms (*P*-value = 0.005).

**Conclusions:**

Overall, mirtazapine seems to have a potential benefit in the treatment of patients with IBS-D, particularly those with concomitant psychological symptoms. However, further studies are warranted to determine whether these findings are replicated.

**Trial registration:**

Trial registration: Registration number at Iranian Registry of Clinical Trials: IRCT20120215009014N311. Registration date: 2019-10-21.

## Background

Irritable bowel syndrome (IBS) is the most common functional gastrointestinal disorder with a relapsing/ remitting course. It is a symptom-based condition defined by abdominal pain and discomfort in association with altered bowel habits, with no identifiable cause [1]. It approximately affects 7–18% of the population worldwide and is more common in women than men [2]. IBS based on patients’ predominant stool pattern clinically is classified into four subtypes: with diarrhea (IBS-D), with constipation (IBS-C), mixed type (IBS-M), and un-subtyped (IBS-U). Determining the subtype of IBS is important for both diagnosis and treatment [3]. Although no decrease in life expectancy attributable to IBS has been observed, it generates a significant burden to both patients and society as a result of direct medical costs, lost productivity, and reduced health-related quality of life [4]. Management of IBS is based upon a multifactorial approach and includes non-pharmacological and pharmacological interventions [5]. Due to the high prevalence of psychiatric disorders in patients with IBS, anxiolytics and antidepressants agents, especially the tricyclic antidepressants (TCAs) and selective serotonin reuptake inhibitors (SSRIs) commonly used for management of IBS symptoms. However, it is believed that the benefits of antidepressants in IBS treatment are not limited only to their anxiolytic and antidepressants effects. They may also have peripheral effects on pain perception, visceral hypersensitivity, and GI motility that could help patients with IBS-D [6]. However, although a number of pharmacological treatments are available for the treatment of IBS, most of the currently available drugs focus on alleviating symptoms rather than targeting the underlying pathophysiology. As a consequence, there is no satisfactory treatment at present for the management of IBS, and vast numbers of patients with IBS experience suboptimal clinical relief from current treatments [7]. Thus, there is a need for alternative effective pharmacological treatment for IBS.

For the development of new effective treatments for IBS, a better understanding of the potential underlying mechanisms involved in the generation of symptoms is crucial. Although, the exact pathophysiology of IBS has not yet been fully elucidated, overall, symptoms appear to be related to alterations in GI motility and/or enhanced visceral sensitivity [8]. Serotonin (5-HT) is an important neurotransmitter and paracrine signaling molecule in the gastrointestinal tract that it’s releasing from enterochromaffin (EC) cells initiates peristaltic, secretory, vasodilatory, vagal, and nociceptive reflexes [9]. A large body of evidence indicates that abnormalities of serotonergic function have a central role in both intestinal and extraintestinal symptoms of IBS [10]. It has been found 5-HT dysfunction in the gut by affecting intestinal motor and secretory function may lead to either constipation or diarrhea. Additionally, altered 5-HT signaling in the central nervous system and the gut contributes to hypersensitivity in IBS [11]. These causative mechanisms suggest that therapies targeting altered serotonin signaling may provide new, effective treatments for patients with IBS. Several serotonin receptor subtypes have been identified that of which the most interesting targets for pharmacological intervention for IBS are 5-HT3 and 5-HT4 receptors subtypes [12].

Around one-third of patients with IBS meet the criteria for IBS-D with common symptoms of abdominal pain or discomfort, frequent loose stools, and urgency [13]. It is well known that 5-HT3 receptor antagonists by diminishing motor and secretory reflex activity and decreasing the activation of extrinsic sensory neurons that transmit signals to the brain can improve symptoms of stool frequency, urgency, abdominal discomfort, and stool consistency in patients with IBS-D [14]. In this view, a recent systematic review and meta-analysis of randomized controlled trials also reported that 5-HT_3_ receptor antagonists are effective for treating non-constipated IBS and IBS-D. However, although rare, there are still clear concerns regarding the occurrence of serious adverse effects such as ischemic colitis with these agents [15]. Thus, the constant search is ongoing to identify new more effective, and better tolerated 5-HT3 receptor antagonists for the management of IBS-D. One strategy is the search on the existing drugs with known 5-HT3 receptor antagonist property that there is extensive clinical experience in their use and their safety profile.

Mirtazapine is an atypical antidepressant drug that exhibits both noradrenergic and serotonergic activity. Mirtazapine promotes the release of noradrenaline and serotonin by blocking α_2_-adrenergic autoreceptors and α_2_-adrenergic heteroreceptors, respectively. It also enhances serotonin neurotransmission, mainly through 5-HT_1A_ receptors by blocking postsynaptic 5-HT_2A_, 5-HT_2C_, and 5-HT_3_ receptors [16]. Since introduction, because of its unique mechanisms of actions, in addition to treating depression and anxiety, the potential usefulness of mirtazapine in the treatment of many other psychiatric and medical conditions has been investigated [17]. Considering the 5-HT3 receptor antagonist property of mirtazapine, it may be also a valuable therapeutic option for the management of patients with IBS-D and preliminary evidence indicates its beneficial effects [18, 19].

However, there are only case reports and open-label studies, and double-blind, placebo-controlled studies with mirtazapine in the treatment of IBS-D are lacking. Hence, we designed this double-blind, placebo-controlled study to evaluate whether compared to placebo, mirtazapine would be efficacious and safe in the treatment of patients suffering from IBS-D.

## Material and methods

### Study design

This was an 8-week double blind, randomized, placebo-controlled study that from November 2019 until July 2020 was conducted in a gastroenterology clinic, at a tertiary referral hospital, affiliated to Hamadan University of Medical Sciences, Hamadan, Iran, to evaluate the efficacy of mirtazapine in the treatment of IBS-D. The trial comprised a one-week run-in period and an 8-week intervention phase which consisted of a 1 week dose titration period followed by a 7-week fixed-dose period. Eligible patients were fully informed about the study aims and all patients signed written informed consent prior to study participation. The trial was performed in accordance with the Declaration of Helsinki and subsequent revisions and approved by the ethics committee at Hamadan University of Medical Sciences. The trial was registered at the Iranian Registry of Clinical Trials (IRCT20120215009014N311).

### Participants

Subjects were men and women age 18–75 years with a diagnosis of IBS with a subtype of diarrhea defined by the Rome IV criteria [20]. To exclude other causes of diarrhea, patients should have normal colonic anatomy (as assessed by flexible sigmoidoscopy, colonoscopy, or double-contrast barium enema plus flexible sigmoidoscopy), normal full blood count, serum calcium and albumin, C-reactive protein, normal thyroid-stimulating hormone levels, negative lactose intolerance test, negative serological tests for celiac disease, and negative stool examinations. During the 1-week run-in period, data on Stool form (appearance) and stool frequency were collected daily to ensure that patients had suitable symptom levels at the start of the study. Stool form data were scored on a 7-point ordinal scale according to the Bristol Stool Form (BSF) Scale [19]. Based on the response to daily stool diary during the 1-week run-in period, patients should experience loose stools for ≥3 days in a week with a Bristol Stool Scale type 6 (fluffy pieces with ragged edges, a mushy stool) or 7 (watery stool, no solid pieces; entirely liquid stool). Exclusion criteria were the following: patients with a diagnosis of IBS with a subtype of constipation, mixed IBS, or un-subtyped IBS by the Rome IV criteria [20], having organic GI disease (e.g., colitis, Crohn’s disease, celiac disease, bowel surgery, recurrent diverticulitis), history of lactose intolerance, any antidepressant treatment in the previous 3 months, consuming any medication that could affect the outcomes in the clinician’s opinion (including anticholinergic medications, antibiotics, pain medications that contained opiates or morphine, 5-HT3 receptor antagonists, diarrhea medication, medication that accelerates the emptying of the stomach, laxatives, cholestyramine, probiotic products, etc.) within at least 7 days before entering the study, unstable medical condition, hyperthyroidism or hypothyroidism, previous gastrointestinal surgery, pregnancy or lactation or expecting to get pregnant during the study, medical or psychological factors interfering with the collection or interpretation of study data, inadequate education and skill for being interviewed and completing questionnaires, history of drug or alcohol abuse, and presence of any adverse effects resulting in patients’ intolerance or complications.

### Intervention

From November 2019 until August 2020, 118 patients with a diagnosis of diarrhea-predominant IBS who attended a gastroenterology clinic at a tertiary referral hospital were assessed for eligibility. Of these, 67 patients who met the inclusion/exclusion criteria and agreed to participate in the study were randomized in a double-blind fashion (using a block size of 4 in a 1:1 ratio) into either the mirtazapine treatment group (intervention group; *n* = 34) or the placebo treatment group (control group; *n* = 33). The randomization was provided by an independent statistician to ensure that groups were matched for age and sex where possible. Both the investigators and the patients were blinded to the treatment.

Patients according to their group allocation were instructed to receive mirtazapine or placebo. Both mirtazapine and placebo tablets were identical in shape, color, and odor; the packaging of the compounds was likewise identical. Patients started with mirtazapine 15 mg/day at bedtime for 1 week; after which the dose was increased to 30 mg/day and was continued with this dose for the entire duration of the study. Placebo was administered in an identical manner. Use of loperamide (up 2 mg three times daily) as a rescue medication was permitted for treatment of acute uncontrolled diarrhea, but the patients were required to not use any loperamide rescue medication during the 1-week run-in period, and during the last week of the treatment period.

Adherence to treatment was determined by counting drugs left in the container at the end of the treatment period and patients were considered adherent to treatment if at least 80% of all doses were taken. The demographic and baseline characteristics of the subjects were recorded at the first visit. Adverse events were also recorded throughout the treatment period through phone calls.

### Efficacy and safety assessment

Outcome measures were assessed using validated questionnaires. All subjects were required to complete daily stool diary on paper diary cards at bedtime for a 7-day period, once during the 1-week run-in period, prior to consuming any treatment, and once during the last week of the treatment. Daily stool diary provides information on stool consistency (based on the Bristol Stool Form Scale (BSFS) from 1 (very hard) to 7 (watery)), frequency of defecation, and the severity of abdominal pain, urgency of defecation, and bloating that the last three scored as none, mild, moderate or severe (0–3) [21]. A number of days with pain, urgency, diarrhea (defined as more than three bowel movements per day), and bloating were also recorded at the 1-week run-in period and the last week of the treatment period. The baseline values of stool consistency scores based on the BSFS, the severity of abdominal pain, the severity of urgency of defecation, the severity of bloating, and the frequency of defecation per day were averages from the 1-week run-in period, and the endpoint values were averages from the last week of the treatment period. Patients were reminded by telephone twice weekly to complete their daily diaries.

Further, once at the end of 1-week run-in period (before starting treatment) and once after 8 weeks of treatment (end of the study period), the subjects were requested to complete following questionnaires: 1) the Hospital Anxiety and Depression Scale questionnaire (HADS) for assessing psychological comorbidities 2) the IBS Severity Scoring System questionnaire (IBS-SSS) for assessing the severity of IBS symptoms, and 3) the IBS Quality of Life (IBS-QoL) questionnaire for assessing the degree to which IBS interferes with patient quality of life. The HADS questionnaire is a self-report measure that was specifically developed to assess anxiety and depression in people with medical illnesses. This questionnaire contains 14 questions with seven items for each subscale of anxiety or depression. Each item is rated on a four-point scale (0–3) and the total score for each subscale of depression or anxiety ranges from 0 (no depression, no anxiety) to 21 (maximal depression or anxiety) [22]. The reliability and validity of the HADS questionnaire in the Iranian population have been assessed by Montazeri et al. [23]. IBS-SSS is a patient based scale that assesses 5 clinically relevant items during a 10-day period: (1) severity of abdominal pain, (2) frequency of abdominal pain, (3) severity of abdominal distention or tightness, (4) dissatisfaction with bowel habits, and (5) interference of IBS with life in general. Each item is scored on a visual analog scale (VAS) from 0 to 100, yielding overall scores ranging from 0 to 500 (a higher score indicates worse condition) [24]. IBS-QoL questionnaire as a 34-items instrument was also developed and validated to assess QOL impairment in IBS. Each item is rated on a 5-point Likert scale where 1 generally represents better responses on items and 5 represents worse responses; thus yielding a total score that has a theoretical range of 34 to 170, with higher scores indicating worse QOL. For ease of interpretation, as per the original description of the tool, the raw scores of the IBS-QoL were converted to a 0–100 scale, with higher scores indicating better IBS specific quality of life [25]. A Persian translated and validated version of this questionnaire was used in this study [26].

Main primary outcome measures in the study were the changes in the total IBS-SSS score from baseline to end of treatment, and the proportion of responders which was defined as number of patients whose overall symptom severity scores on the IBS-SSS changed ≥50 points from baseline to Week 8 [24, 27]. As secondary efficacy outcomes, the changes in severity of the five sub-scales of IBS-SSS, the above mentioned diary-based symptoms scores including pain perception, urgency of defecation, bloating, stool frequency defined as a mean number of episodes per day, stool consistency using the 7-point BSFS, and a number of days per week with pain, urgency, diarrhea, or bloating were reported. Additional efficacy assessment outcomes included the changes in the IBS-QoL and HADS total scores at the end of the treatment period compared to the baseline. Also, as another secondary outcome, all randomized patients in the intention-to-treat (ITT) population who received at least one dose of study medication were evaluated for safety.

### Statistical analyses

All analyses were performed on an ITT analysis data set because all participants received at least one dose of the study medication. Because dropout rates of less than 20% and similar courses of disease in the comparison groups, the missing data were replaced by the mean data of the other group. Data were analyzed using SPSS for Windows (SPSS Inc., Chicago, IL, USA) version 16 software. The Kolmogorov-Smirnov test was performed to evaluate the normality of the continuous variable distribution. Normally distributed continuous variables were reported as mean ± standard deviation (SD). Non-normally distributed continuous data were expressed as (interquartile range [IQR]). Categorical variables were reported as frequency and percentage. Parametric and non-parametric continuous variables were analyzed using independent *t*-test and Mann–Whitney tests, respectively. The distribution of categorical variables between two groups was compared using the Chi-square or Fisher exact test (if more than 20% of the categories were expected to have frequencies less than 5). A two-tailed *P*-value less than 0.05 was considered statistically significant.

## Results

### Participant flow

**Fig. 1:**
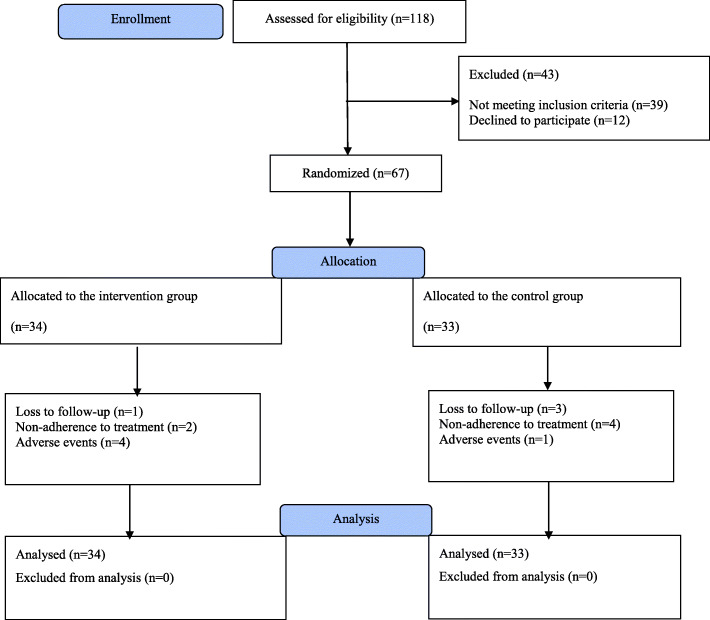
The flow diagram of the study

Figure 1 shows the flow diagram of the trial participants. A total of 118 patients with a diagnosis of IBS with a subtype of diarrhea underwent a screening examination to ensure study eligibility. Of those, 12 patients did not agree to take part in the study and 39 patients did not meet the inclusion/exclusion criteria at baseline. The remaining 67 patients who met the study criteria and had an interest in study participation were enrolled in the study (34 patients in the mirtazapine-treated group and 33 patients in the placebo-treated group). Of whom, 54 patients (80.6%) completed the entire course of the study and 13 patients (19.4%) discontinued participation in the study prematurely: 5 patients due to experiencing intolerable adverse effects, 4 patients due to loss to follow up, and 4 patients due to using the medication for less than 80% of the study period. As mentioned above, all analyses were performed on an ITT analysis data set (on 67 patients).

### Demographics and baseline characteristics

The basic characteristics of the study population are depicted in Table 1. Of the included patients, 32.8% (22 patients) were male and 67.2% (45 patients) were female. The gender distribution was in favor of females in both groups (72.7% in the control group and 61.8% in the intervention group). The mean age (±SD) of the patients in the mirtazapine and placebo groups was 44.41 (±11.25) and 43.45 (±10.35) years, respectively. At baseline, the mean values of the Body Mass Index (BMI) were 22.27 ± 2.95 and 22.71 ± 3.19 in the mirtazapine and placebo groups, respectively. The mean duration of IBS was 10.0 ± 6.52 years (ranged from 1 to 25 years) in the mirtazapine group and 9.2 ± 7.74 years (ranged from 1 to 28 years) in the placebo group. As showed in Table 1, the treatment groups were comparable with regards to basic characteristics including age, gender, BMI, and mean duration of illness.

**Table 1:** Baseline demographic and clinical characteristics of the patients

Variable	Groups		*P* value
	Control (*N* = 33)	Intervention (*N* = 34)	
Gender (N; male/female)	9/24	13/21	0.44
Age (years; mean ± SD)	43.45 ± 10.35	44.41 ± 11.25	0.95
BMI (Kg/m^2^; mean ± SD)	22.71 ± 3.19	22.27 ± 2.95	0.57
IBS duration (Years; mean ± SD)	9.2 ± 7.74	10.0 ± 6.52	0.65

*BMI* Body mass index, *IBS* Irritable bowel syndrome

### Efficacy outcomes

**Fig. 2:**
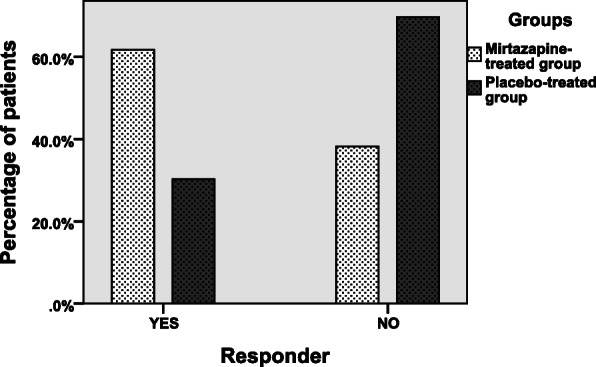
Comparison of responder rates between two groups, as defined by a 50-point or more reduction in IBS-SSS overall score at the end of the study period (*P*-value = 0.01)

Table 2 shows the change in the clinical outcomes including IBS-SSS, IBS-QoL, and HADS scores from baseline to week 8 by treatment allocation. At baseline, the study groups were similar with respect to the total IBS-SSS score (301.68 ± 78.73 in the mirtazapine-treated subjects and 294.09 ± 71.55 in the placebo-treated subjects; *P*-value = 0.68) (Table 2). Although in both treatment groups, mean total IBS-SSS scores declined from baseline to the end of the intervention, the change in IBS-SSS overall score during the trial course was significantly greater in the mirtazapine-treated subjects than the placebo-treated subjects (− 89.76 ± 71.60 vs. -34.73 ± 66.91; *P*-value = 0.002) (Table 2). In more statistical details, the five sub-scales of IBS-SSS were also analyzed. When compared to the placebo group, except for the score for severity of abdominal distention, eight-week change in all other IBS-SSS sub-scale scores were significantly greater in the mirtazapine group (*P*-value<.05 for all, with the exception of abdominal distention; Table 2). With respect to responder rate which was defined as a 50-point or more reduction in IBS-SSS overall score at the end of treatment, 61.8% (21 out of 34 patients) in the mirtazapine-treated group compared to 30.3% (10 out of 33 patients) in the placebo-treated group were responders, which was a statistically significant difference (*P*-value = 0.01; Fig. 2).

**Table 2:** Results related to the changes of IBS-SSS total score, IBS-SSS sub-scale scores, IBS-QoL score, and HADS score over time in the two groups

Variable	Group	Baseline	Week 8	Mean difference
Total IBS-SSS score	Intervention	301.68 ± 78.73	211.92 ± 62.72	− 89.76 ± 71.60
	Control	294.09 ± 71.55	259.36 ± 72.81	− 34.73 ± 66.91
	*P*- value	0.68	**0.006**	**0.002**
IBS-SSS Pain severity score	Intervention	68.53 ± 18.93	41.47 ± 17.08	− 27.06 ± 18.83
	Control	61.18 ± 15.71	52.53 ± 16.40	−9.00 ± 14.87
	*P*- value	0.10	**0.009**	**< 0.001**
IBS-SSS Pain frequency score	Intervention	60.59 ± 20.59	42.06 ± 19.81	−18.52 ± 16.54
	Control	60.24 ± 14.06	54.21 ± 14.18	−6.03 ± 15.82
	*P*- value	0.94	**0.005**	**0.002**
IBS-SSS abdominal distention severity score	Intervention	61.76 ± 22.35	50.29 ± 18.50	−11.47 ± 12.34
	Control	61.43 ± 16.96	53.58 ± 16.86	−7.84 ± 16.41
	*P*- value	0.91	0.45	0.31
IBS-SSS bowel habit dissatisfaction score	Intervention	58.53 ± 22.85	39.41 ± 15.94	−19.12 ± 21.08
	Control	58.06 ± 18.03	51.21 ± 17.98	−6.97 ± 14.68
	*P*- value	0.84	**0.006**	**0.008**
IBS-SSS life interference score	Intervention	51.47 ± 21.90	37.65 ± 18.60	−13.82 ± 15.18
	Control	54.09 ± 18.69	48.79 ± 18.50	−5.30 ± 12.12
	*P*- value	0.60	**0.01**	**0.02**
HADS-A	Intervention	9.36 ± 3.26	5.57 ± 1.89	−3.70 ± 3.48
	Control	8.88 ± 2.33	7.19 ± 2.65	−1.69 ± 2.79
	*P*- value	0.59	**0.005**	**0.01**
HADS-D	Intervention	8.15 ± 2.99	5.97 ± 1.58	−2.18 ± 3.74
	Control	7.73 ± 2.77	6.88 ± 2.52	−0.85 ± 3.23
	*P*- value	0.55	0.08	0.13
IBS-QoL	Intervention	45.32 ± 17.18	70.52 ± 16.55	25.19 ± 15.60
	Control	53.11 ± 16.34	62.68 ± 14.25	9.56 ± 14.89
	*P*- value	0.06	**0.04**	**< 0.001**

*IBS-SSS* IBS Severity Scoring System, *IBS-QoL* IBS Quality of Life, *HADS* Hospital Anxiety and Depression Scale questionnaire

Results regarding the changes in the diary-based symptoms in the two groups have been reported in Table 3. As results show, compared to the baseline at the end of the treatment period all diary-derived symptoms except bloating showed significantly more improvement in the mirtazapine-treated subjects compared to the placebo-treated subjects. The median (IQR) of average stool consistency score based on the BSFS score decreased from 5.50 (IQR: 6–5.07) (at the week prior to starting treatment of the trial) to 4.00 (IQR: 4.5–3.5) (at the last week of trial) in the mirtazapine-treated group, and decreased from 5.35 (IQR: 5.8–5) to 5.00 (IQR: 5.5–4.5) in the placebo-treated group. The median (IQR) of change in the average stool consistency score in the mirtazapine group was − 1.25 (IQR: − 0.8-(− 1.9)) and in the placebo group was − 0.30 (IQR: 0.2-(− 0.6)), which showed a statistically significant difference between the two groups (*P*-value < 0.001). Further, mirtazapine reduced the mean average daily bowel movement frequency from 2.30 ± 0.69 (at the week prior to starting treatment of the trial) to 1.83 ± 0.43 (at the last week of trial) which compared to placebo was a significantly greater reduction. Likewise, in the last week of the treatment period, with regard to the mean average urgency or abdominal pain scores as well as the median number of days with abdominal pain, urgency, and diarrhea, more favorable outcomes were observed in the mirtazapine-treated subjects compared to the placebo-treated subjects.

**Table 3:** Results related to the changes in the diary-based symptoms in the study groups

Variable	Group	Baseline	Week 8	Mean difference
BSFS Median,(IQR)	Intervention	5.50 (6–5.07)	4.00 (4.5–3.5)	−1.25(− 0.8-(− 1.9))
	Control	5.35 (5.8–5)	5.00 (5.5–4.5)	− 0.30 (0.2-(− 0.6))
	*P*- value	0.34	**< 0.001**	**< 0.001**
Bowel frequency per day Mean ± SD	Intervention	2.30 ± 0.69	1.83 ± 0.43	− 0.48 ± 0.41
	Control	2.27 ± 0.63	2.15 ± 0.57	− 0.12 ± 0.31
	*P*- value	0.79	**0.01**	**< 0.001**
Abdominal pain score Mean ± SD	Intervention	1.50 ± 0.45	0.97 ± 0.45	− 0.54 ± 0.41
	Control	1.65 ± 0.62	1.44 ± 0.58	− 0.21 ± 0.37
	*P*- value	0.27	**< 0.001**	**0.001**
Urgency score Mean ± SD	Intervention	1.70 ± 0.48	1.13 ± 0.47	− 0.57 ± 0.33
	Control	1.57 ± 0.57	1.41 ± 0.53	− 0.16 ± 0.29
	*P*- value	0.31	**0.03**	**< 0.001**
Bloating score Mean ± SD	Intervention	1.61 ± 0.57	1.38 ± 0.39	− 0.25 ± 0.43
	Control	1.51 ± 0.57	1.32 ± 0.50	− 0.19 ± 0.35
	*P*- value	0.47	0.69	0.54
Days with urgency Median,(IQR)	Intervention	5.00 (6–4)	4.00 (5–3)	− 2.00 (0.00- (− 2))
	Control	6.00 (6–4)	5.00 (6–3)	0.00 (0.00- (− 2))
	*P*- value	0.36	**0.01**	**0.05**
Days with pain Median,(IQR)	Intervention	5.00 (6–4)	3.00 (4–2)	− 2.00 (− 1-(− 2))
	Control	5.00 (5.5–4)	5.00 (5–3.5)	0.00 (0.00- (− 1))
	*P*- value	0.75	**< 0.001**	**< 0.001**
Days with diarrhea Median,(IQR)	Intervention	5.00 (6–4)	4.00 (4–2.75)	− 1.50 (0.75- (− 2.25))
	Control	5.00 (6–4)	4.00 (5–4)	0.00 (0.00- (− 2))
	*P*- value	0.84	**0.001**	**0.003**
Days with bloating Median,(IQR)	Intervention	6.00 (7–5)	5.00 (6–3.75)	− 1.00 (0.00- (− 2))
	Control	5.00 (7–4.5)	5.00 (6–4)	− 1.00 (1- (− 1.5))
	*P*- value	0.59	0.17	0.11

*BSFS* Bristol Stool Form scale, *IQR* Interquartile range, *SD* Standard deviation

With regard to the patients’ anxiety symptoms, at the end of the study period, we noted more improvement in the mirtazapine-treated subjects compared to the placebo-treated subjects (the mean HADS-A scores decreased from 9.36 ± 3.26 to 5.57 ± 1.89 in the mirtazapine group and decreased from 8.88 ± 2.33 to 7.19 ± 2.65 in the placebo group; *P*-value = 0.005; Table 2). Although at the end of the treatment period, depression symptoms also showed more improvement in the mirtazapine-treated subjects compared to the placebo-treated subjects, it did not display significant differences. Concerning the impact of IBS on patients’ quality of life, at the end of the treatment period, a noticeable improvement in the IBS-QoL score was also seen in the intervention group compared to the control group (the IBS-QoL score increased from 45.32 ± 17.18 at baseline to 70.52 ± 16.55 on week 8 after treatment in the patients receiving mirtazapine and from 53.11 ± 16.34 to 62.68 ± 14.25 in the patients receiving placebo; *P*-value = 0.04; Table 2).

### Safety outcomes

Table 4 demonstrates the frequency of drug-related adverse effects by treatment allocation. As shown, adverse effects such as drowsiness, dry mouth, fatigue, and increased appetite were reported by a higher percentage of the mirtazapine-treated subjects compared to the placebo-treated subjects. Nevertheless, as these adverse effects were mild to moderate in nature, mirtazapine was acceptably well-tolerated by a considerable percentage of the study patients and only 4 patients in the mirtazapine group discontinued their treatment due to intolerable adverse effects. Further, during the treatment period, 8 patients in the mirtazapine group and one patient in the placebo group gained weight (*P*-value = 0.03) (between 2 and 5 kg, self-reported) which could be advantageous in the patients with anorexia.

**Table 4:** Frequency of drug related adverse effects among patients in each group

Adverse effects N (%)	Group		***P***- value
	Mirtazapine (***N*** = 34)	Placebo (***N*** = 33)	
Drowsiness	12 (35.3%)	3 (9.1%)	**0.01**
Dizziness	6 (17.6%)	2 (6.1%)	0.25
Nausea	5 (14.7%)	2 (6.1%)	0.43
Dry mouth	9 (26.5%)	2 (6.1%)	**0.04**
Fatigue	10 (29.4%)	2 (6.1%)	**0.02**
Headache	4 (11.8%)	1 (3.0%)	0.36
Increased appetite	11 (32.4%)	2 (6.1%)	**0.01**
Weight gain	8 (23.5%)	1 (3.0%)	**0.03**

## Discussion

Based on our best knowledge, our study for the first time in a randomized, double-blind, and placebo-controlled study evaluated the influence of mirtazapine on clinical outcomes of patients with diarrhea-predominant IBS. This study showed that in comparison to the placebo, mirtazapine was more effective in decreasing the severity of IBS symptoms (based on the IBS-SSS questionnaire). Further, compared to placebo, mirtazapine increased stool consistency, decreased stool frequency, decreased urgency and abdominal pain scores, and increased the rate of days without bowel urgency, pain, and diarrhea. Additionally, while was acceptably well-tolerated, treatment with mirtazapine improved the patients’ quality of life as well as psychological symptoms such as anxiety.

Diarrhea-predominant IBS is a debilitating form of IBS which significantly reduces the quality of life of patients, increases health care expenditures, impairs social and occupational functioning, and increases psychological disorders [28]. Despite its high burden, the choice of treatments of IBS-D by patients or their practitioners is still challenging. Although a number of different pharmacological treatments are available for the management of patients with IBS-D, no treatment has been shown to have sufficient efficacy and safety and most of the patients continue to suffer from symptoms [29]. The pathophysiology of IBS is not precisely understood yet and likely it has multifactorial etiopathogenesis. From the biological point of view, the coordination between the central nervous system and gastrointestinal contractility is regulated through a variety of brain-gut peptides and gastrointestinal hormones [30]. Among a variety of neurotransmitters that can evoke IBS symptoms, serotonin has key contributory roles in its pathogenesis. Serotonin is an important signaling molecule in the activation of motor and secretory reflexes, and in the activation of sensory signals in the brain-gut axis. It is becoming increasingly clear that dysfunctions of the central or peripheral serotonergic system can be involved in the pathophysiology of IBS [15]. In this context, it has been reported that patients with IBS-D might have reduced serotonin reuptake, and those with IBS-C might have impaired release of serotonin [31]. Further studies have also indicated that post-infective IBS is associated with increased serotonin containing enteroendocrine cells [32] and increased postprandial serotonin release [33]. There is also evidence of an association between serotonin transporter gene polymorphism and the diarrhea-dominant IBS phenotype. Indeed, it seems that the reduction of serotonin transporter expression could result in raised serotonin levels and contribute to symptoms in patients with IBS-D [34]. Among multiple subtypes of the receptor for serotonin, it is well recognized that the 5HT3 subtype plays an important role in gut function. It has been also known that signaling from the gut to the central nervous system is predominantly 5-HT3 mediated [35]. Further, it is well-known that in diarrhea-predominant IBS, activation of serotonin receptors belonging to the 5-HT3 subtype by increasing the firing rate of secretomotor neurons increase intestinal motility and secretion [36]. In line with this evidence, it is found that 5-HT3-receptor antagonists retard colonic transit, reduce secretion, reduce visceral sensitivity via both peripheral and central nervous receptors system mechanisms, and increase colonic compliance in response to distension [37]. Actually, it is found that the 5-HT3 receptor antagonists by inhibiting 5-HT_3_ receptors located on intrinsic sensory neurons can diminish motor and secretory reflex activity, and by decreasing the activation of extrinsic sensory neurons and vagal afferents, which are involved in the transmission of noxious and non-noxious (eg, nausea, bloating) sensations respectively, decrease the visceral pain and discomfort associated with IBS [38]. So, this evidence makes 5-HT3 antagonists as a logical treatment in the management of patients with IBS-D. In line with this, a recent systematic review and meta-analysis by Zheng et al. showed that 5-HT_3_ receptor antagonists relieve global IBS-D symptoms, abdominal pain and discomfort, urgency, stool consistency, and stool frequency in non-constipated IBS and IBS-D [15]. However, despite the substantial evidence of their effectiveness, due to concern regarding occurrence rare but serious adverse events such as severe constipation and ischemic colitis, place of 5-HT_3_ receptor antagonists such as cilansetron and alosetron in the treatment of IBS-D being restricted to patients with severe refractory IBS-D who have failed to respond to the usual conventional treatment [35]. Therefore, there is a need for alternative safe and efficacious pharmacological treatment for IBS-D. It seems that instead of new drug discovery, attempts to introduce agents with 5-HT3 receptor antagonist property from already-existing drugs with known adverse drug reaction profiles may be a good strategy.

One of these agents is mirtazapine. Mirtazapine is an antidepressant and antianxiety agent that because of the unique mechanism of action, since introduction, its use in a wide range of conditions has been investigated [16]. Recently, some clinical studies also have addressed its effectiveness in the management of functional gastrointestinal symptoms. In this regard, Jiang et al. in their recent study in functional dyspepsia patients with weight loss reported that mirtazapine not only alleviates symptoms associated with dyspepsia and depression but also significantly increases body weight. They also observed that the clinical efficacy of mirtazapine may be mediated in part through the regulation of brain-gut or gastrointestinal hormones [39]. Similar to these findings, results of another study by Tack et al. also showed that compared to placebo mirtazapine at a dose of 15 mg/day can improve early satiation scores and nutrient tolerance in functional dyspepsia patients with weight loss [40]. Recently by Sanagapalli and colleagues in an open-label study, the efficacy of mirtazapine in the treatment of IBS-D was also investigated. Consistent with our findings, in this study mirtazapine demonstrated considerable beneficial effects on both gastrointestinal and psychological symptoms in patients with IBS-D [18]. Also, in one case report, mirtazapine was an effective agent in the improvement of debilitating IBS symptoms in a woman that mirtazapine was initiated by her psychiatrist for treatment of comorbid depression [19]. Spiegel et al. also reported mirtazapine was effective in improving both psychopathological symptoms and diarrhea and constipation symptoms in a 66-year-old woman suffering from a 1-year history of IBS-mixed type [41].

Although the beneficial effects of mirtazapine in the treatment of IBS-D mainly stem from its strong antagonistic activity against central and peripheral 5HT3 receptors [42], it seems that mirtazapine has a number of other possible mechanisms of action in the treatment of IBS-D. There is a large body of evidence suggesting that mast cell activation which contains granules rich in mediators such as histamine plays an important role in the development of major IBS symptoms, such as abdominal pain, constipation, and diarrhea [43]. High levels of histamine were found from supernatants from IBS colonic samples [44]. Moreover, it has been observed that the expression of histamine1 (H1) and H2 receptors in the intestinal tissue samples of IBS patients is up-regulated [45]. It is found that histamine by activation enteric neurons through H1 and H2 receptors contributes to visceral hypersensitivity [46]. This causative mechanism suggests the possible application of anti-histamine agents as a potential therapeutic option in the management of IBS and interestingly enough, some experimental and clinical studies have provided promising evidence regarding the effectiveness of these agents in the treatment of IBS [47–49]. Mirtazapine is also found to have a high affinity for central and peripheral H1receptors and acts as a potent antagonist of H1 receptors [50]. So, it seems that the anti-histamine property of mirtazapine may be another potential mechanism of its action for treating IBS.

Recently accumulated experimental and clinical evidence has reported that mirtazapine like TCAs, as a dual-acting antidepressant, has also marked antinociceptive effects that offer this possibility that mirtazapine used in the management of various chronic pain conditions [51–54]. As regards the mechanism of action, it is believed the antinociceptive effect of mirtazapine unrelated to its antidepressant and antianxiety effects. It seems mirtazapine mainly via modulation of serotonin and noradrenaline pathways in the brain, and selective interaction with multiple 5-HT receptors exerts its antinociceptive effects [54, 55]. Beyond this, in recent years mounting of evidence has pointed out that Kappa opioid receptors which are located on the terminals of a variety of neurons, including those extrinsic visceral afferent neurons exhibit visceral analgesic and antihyperalgesic activity [56, 57]. Preliminary evidence suggests that beside serotonergic and noradrenergic receptors, the antinociceptive effects of mirtazapine is partially mediated by the opioidergic system, particularly through activation of the kappa3-opioid receptor subtype [54, 55].

It is well-known that psychological stress playing a major role in the onset and exacerbation of IBS symptoms such as abdominal pain and altered bowel movements [58]. Considering that psychiatric comorbidities such as anxiety and depression are highly prevalent in IBS patients [59], and psychopharmacological agents commonly employed by clinicians as an alternative therapy for the management of IBS symptoms, especially IBS-D [6], it seems that the anxiolytic and antidepressant effect of mirtazapine is an additional mechanism that may indirectly through it alleviate IBS symptoms.

Taken together, considering the above-discussed mirtazapine ways of action, it can exert beneficial effects on IBS symptoms via multiple potential mechanisms of action. Interestingly enough benefits of mirtazapine treatment in patients with IBS-D are amplified by the fact that it is effective as both an etiological and symptomatic treatment in these patients. Further, the attractiveness of mirtazapine for the treatment of IBS also stems from its good tolerability and safety profile. Dry mouth, sedation, and weight gain are the most common side effects of mirtazapine that tolerance to side effects such as sedation can develop with ongoing therapy [16]. Further, its appetite stimulation may be welcome in patients with anorexia and low BMI [39, 40]. Evidence concerning the effect of mirtazapine on muscarinic-cholinergic receptors is limited. Although previously, some experimental evidence indicated that mirtazapine has a low affinity for central and peripheral muscarinic-cholinergic receptors [60, 61], recently preliminary evidence suggests that mirtazapine interacts with muscle and neuronal muscarinic and nicotinic receptors as well [62, 63]. However, the clinical importance of the anticholinergic effect of mirtazapine especially in a matter of its tolerability remains unclear. However, it is obviously clear that Mirtazapine particularly in the case of anticholinergic side effects generally better tolerated than the traditional TCAs that are routinely used in the management of IBS symptoms [64]. Therefore, this margin of safety and excellent efficacy makes mirtazapine as a promising agent for the management of IBS-D.

Despite the novelty, the findings of our work should be interpreted within its limitations. The first limitation of this study is the relatively small number of subjects and the relatively short duration of the follow up that can affect the generalization of our results. More studies with larger sample sizes and longer follow-up visits are warranted to validate the findings reported here. The second limitation of the study is that the patients in our study received a relatively modest dose of mirtazapine; maybe higher doses show better effects on the improvement of symptoms or different tolerability. Third, for the assessment of the rate of occurrence of adverse effects, we relied on the patients’ self-reporting. Since patients may not recognize all drug-related side effects, thus the actual incidence of side effects may be underestimated in this study. Hence, using a standard instrument to identify adverse effects in advance in future studies can estimate more precisely the incidence of adverse effects. Last but not least, we investigate the effectiveness of mirtazapine in patients with diarrhea-dominant IBS. Additional trials are needed to evaluate the effectiveness of mirtazapine for the treatment of other types of IBS.

## Conclusion

In conclusion, compared to placebo, mirtazapine not only alleviated gastrointestinal symptoms of patients with IBS-D but also improved QOL as well as psychological symptoms such as anxiety of these patients. Despite the limitations of our study that make a drawing strong conclusion difficult, our study provides primary evidence that patients suffering from IBS-D symptoms, particularly those with concomitant psychological symptoms, by the administration of mirtazapine experience significant improvement in gastrointestinal symptoms of IBS-D but also in psychological aspects and QOL, as do traditional TCAs. Given that mirtazapine is generally better tolerated than TCAs, this evidence makes mirtazapine an attractive therapeutic option in the treatment of IBS. However, future studies with larger sample size and longer follow-up periods would be necessary to clarify the optimal dose as well as the safety and efficacy of mirtazapine in patients with other subtypes of IBS.

## Data Availability

The datasets used and analyzed during the current study are available from the corresponding author on reasonable request up to 2 years after publication.

## Abbreviations

IBS: Irritable bowel syndrome
IBS-D: Irritable bowel syndrome with diarrhea
IBS-C: Irritable bowel syndrome with constipation
IBS-M: Irritable bowel syndrome mixed type
IBS-U: Irritable bowel syndrome un-subtyped
TCAs: Tricyclic antidepressants
SSRIs: Selective serotonin reuptake inhibitors
5-HT: Serotonin
EC: Enterochromaffin
BSF: Bristol Stool Form
BSFS: Bristol Stool Form Scale
HADS: Hospital Anxiety and Depression Scale questionnaire
IBS-SSS: IBS Severity Scoring System questionnaire
IBS-QoL: IBS Quality of Life
ITT: Intention-to-Treat
SD: Standard deviation
BMI: Body Mass Index
H1: Histamine1
H2: Histamine2
